# Patient‐Reported Outcomes After Treatment With OnabotulinumtoxinA for Platysma Prominence: Results From a Phase 2 Dose‐Ranging Study

**DOI:** 10.1111/jocd.70821

**Published:** 2026-04-03

**Authors:** Shannon Humphrey, Sandhya Shimoga, Joely Kaufman, David Bank, Brian Biesman, Christy Harutunian, Grace S. West, Warren Tong, Vaishali Patel

**Affiliations:** ^1^ Humphrey Cosmetic Dermatology Vancouver British Columbia Canada; ^2^ AbbVie Irvine California USA; ^3^ Skin Associates of South Florida Coral Gables Florida USA; ^4^ The Center for Dermatology, Cosmetic & Laser Surgery Mount Kisco New York USA; ^5^ Private Practice Nashville Tennessee USA; ^6^ Allergan Aesthetics, an AbbVie company Irvine California USA

**Keywords:** botulinum toxin A, cosmetic techniques, neck, patient satisfaction, skin aging, treatment outcome

## Abstract

**Background:**

Repetitive contractions of the platysma muscle may result in vertical neck bands that blunt the jawline (i.e., platysma prominence [PP]). We report patient‐reported outcomes (PROs) from a phase 2 study evaluating onabotulinumtoxinA (onabotA) for moderate or severe PP.

**Methods:**

Subjects were randomized 1:1:1 to onabotA dose 1 (26 U, 31 U, or 36 U), onabotA dose 2 (52 U, 62 U, or 72 U), or placebo. De novo, validated, fit‐for‐purpose PROs were used to assess treatment satisfaction, bother with vertical neck bands and jawline, jawline definition, and psychosocial impact.

**Results:**

Subjects with PRO data (*N* = 164; mean age, 50.0 years) were predominantly female (95.1%) and White (93.9%). Response rates for improvement in both onabotA dose groups were higher than placebo for treatment satisfaction, bother with vertical neck bands and jawline, as well as jawline definition (unadjusted *p* < 0.05 for all). Greater improvements in treatment satisfaction and psychosocial impact were observed in both dose groups versus placebo based on the mean total scores on the Appearance of Neck and Lower Face Questionnaire (ANLFQ): Satisfaction and the mean change from baseline in ANLFQ: Impacts total score, respectively (unadjusted *p* < 0.0001 for all). Response rates generally peaked across all measures at day 14 or 30 for both dose groups, with a trend in improvement continuing through day 120.

**Conclusions:**

Compared with placebo, both onabotA dose groups reported greater satisfaction with the effect of treatment, improvements in jawline definition and psychological impact, as well as reductions in bother due to vertical neck bands and jawline.

**Trial Registration:**

ClinicalTrials.gov: NCT03915067

## 1 Introduction

The platysma muscle is the largest and most inferior mimetic muscle of the face and neck. The platysma muscle complex is composed of 2 separate, superficial muscle sheets that originate from the fascia of the pectoralis muscle below the clavicle and pass upward over both sides of the neck. The size and coverage of the platysma make it the foundation of anterior neck aesthetics, and as such it is the principal target of treatment for an aging neck [1, 2].

Platysma prominence (PP) is a conspicuous characteristic of neck aging that manifests as the loss of jawline definition in the presence of vertical neck bands (i.e., thickened vertical pleats originating in the submandibular area and extending toward the clavicle). PP can occur along with other age‐related features, such as skin laxity (i.e., turkey neck), jowls, and excessive submental fat (i.e., double chin) [1]. PP can become more evident with contraction, such as during speech or smiling, resulting in an aged appearance [3].

Formation of vertical neck bands may be attributed to hyperkinetic platysma activity, making them amenable to treatment with the neuromodulator botulinum toxin A [1, 2]. A recent phase 2 study (NCT04949399) investigating the safety and efficacy of a single treatment of 26 U, 31 U, or 36 U (dose 1) or 52 U, 62 U, or 72 U (dose 2) onabotulinumtoxinA (onabotA) versus placebo in subjects with moderate to severe PP reported statistically significant and clinically meaningful investigator‐ and subject‐rated improvement at day 14 (primary and secondary endpoints, respectively) in the appearance of PP after treatment with onabotA [4].

The prominence in the appearance of the platysma muscle can be aesthetically unappealing, with negative effects on an individual's psychological and emotional well‐being [5]. Individuals with PP may feel that they look older than their actual age, stressed, tired, angry, or less attractive, and may have feelings of self‐consciousness, shame, and insecurity about the appearance of their neck. These feelings may negatively impact their professional and personal lives, including romantic relationships and self‐image, among other aspects, which may motivate patients to seek clinical treatment.

Here, we report results from the same study for the secondary or exploratory patient‐reported outcomes (PROs), which comprehensively assess subjects' treatment experiences based on aspects that are important to the patients, such as treatment satisfaction, bother with vertical neck bands and jawline appearance, jawline definition, and psychosocial impact. These PROs complement the clinician‐ and patient‐reported outcomes of improvements in severity that were published previously [4].

## 2 Methods

### 2.1 Study Design

The study design and methods, including details of the inclusion and exclusion criteria and primary and secondary efficacy endpoints, have been described elsewhere [4]. The 4‐month, multicenter, randomized, double‐blind, placebo‐controlled, parallel‐group, dose‐ranging phase 2 study was conducted from April 23, 2019 to April 16, 2020. Eligible subjects were adults aged ≥ 18 years with moderate (grade 3) or severe (grade 4) PP at maximum contraction on left and right sides at screening and on day 1 as assessed by the investigator using the Clinician Allergan Platysma Prominence Scale (C‐APPS) and by the subject using the Participant Allergan Platysma Prominence Scale (P‐APPS). Both C‐APPS and P‐APPS are scales developed with clinician input and direct input from individuals affected by platysma prominence. They are validated 5‐grade photonumeric scales assessing PP severity (1 = minimal, 2 = mild, 3 = moderate, 4 = severe, 5 = extreme) [4, 6]. Subjects were randomized 1:1:1 to receive a single treatment of onabotA (Botox Cosmetic; Allergan Aesthetics, an AbbVie company, Irvine, CA, USA) dose 1 (26 U, 31 U, or 36 U), dose 2 (52 U, 62 U, or 72 U), or placebo. Subjects were randomized using central by block stratified randomization, wherein baseline C‐APPS grade was used for stratification. Study visits included screening (day −14 to day −7), randomization/study treatment (day 1), follow‐up visits (days 7, 14, 30, 60, and 90), and study exit (day 120). Subject‐reported adverse events (AEs) were recorded at each study visit.

The study was approved by an institutional review board (WIRB Copernicus Group [CGIRG], Cary, NC, USA) and was conducted in accordance with the International Council on Harmonization guidelines for Good Clinical Practice and the Council for International Organizations of Medical Sciences (CIOMS) Ethical Guidelines. All subjects or their legally authorized representatives provided written informed consent.

### 2.2 Dosing Strategy and Injection Technique

**FIGURE 1:**
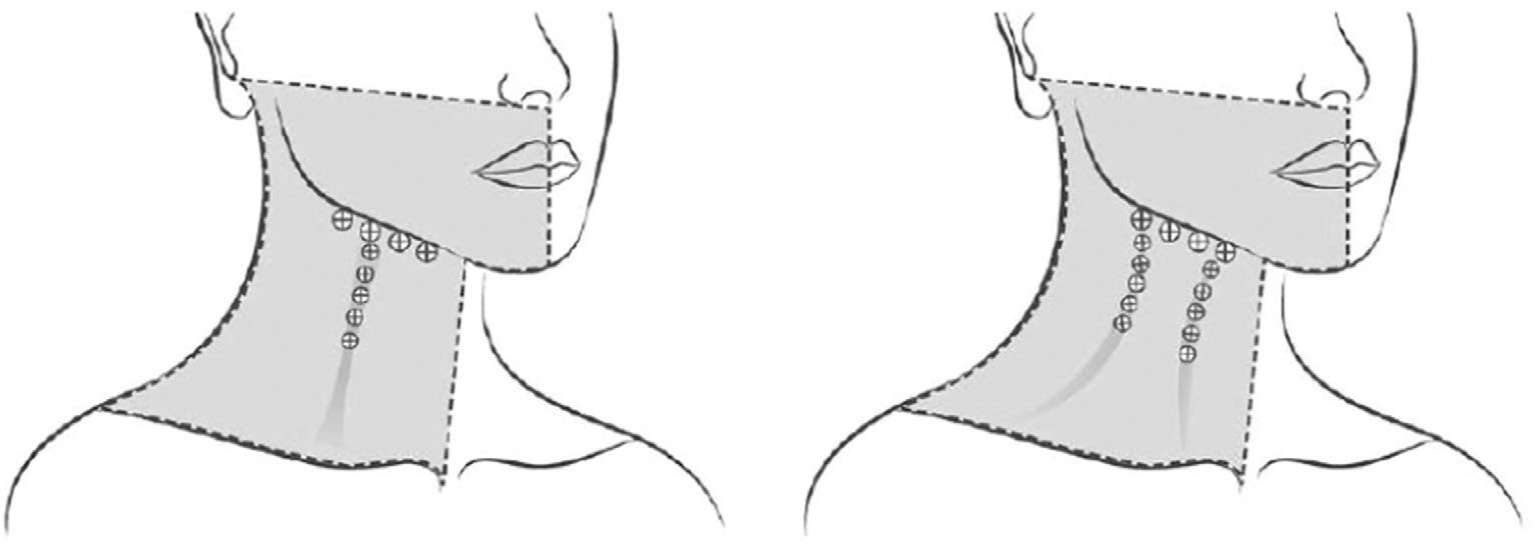
Injection sites based on baseline Clinician Allergan Platysma Prominence Scale (C‐APPS) score of grade 3 (left) or grade 4 (right). Reprinted with permission from Rohrich et al. [4].

As previously published [4], subjects in the dose 1 group received 2 U per injection into 4 sites along the jawline on each side of the face and 1 U per injection into 5 sites on each vertical neck band (Figure 1). Depending on PP severity at baseline, these subjects received either a total of 26 U (1 band on both sides of the face), 31 U (1 band on 1 side of the face and 2 bands on the other side), or 36 U (2 bands per side) of onabotA. Subjects in the dose 2 group received 4 U per injection into 4 sites along the jawline on each side of the face and 2 U per injection into 5 sites on each vertical neck band. Depending on PP severity at baseline, these subjects received either a total of 52 U (1 band per side), 62 U (1 band on 1 side of the face and 2 bands on the other side), or 72 U (2 bands per side) of onabotA. Treatments were administered via superficial intramuscular injections.

### 2.3 PRO Assessments

In addition to severity assessments, the phase 2 study comprehensively assessed concepts that were important and relevant to the individuals affected by the prominence of their platysma muscle. These concepts were derived directly from those individuals via multiple rounds of qualitative interviews and were used to develop PROs. These PROs were further validated and translated in accordance with international regulatory and professional society standards [7, 8]. The concepts captured by the PROs included satisfaction with the effect of treatment, bother with vertical neck bands and jawline appearance, definition of jawline, and psychosocial impact of PP.

The subjects' experiences regarding these concepts were evaluated using the following PROs: Appearance of Neck and Lower Face Questionnaire (ANLFQ): Satisfaction (Baseline and Follow‐up), Bother Assessment Scale for Platysma Prominence (BAS‐PP, formerly called Participant Global Impression of Bother [PGIB]), Participant Global Impression of Severity‐Jawline (PGIS‐Jawline), and ANLFQ: Impacts [9]. The ANLFQ: Satisfaction (Baseline) is a 7‐item measure that includes the Treatment Expectations and Condition‐related Satisfaction domains. The ANLFQ: Satisfaction (Follow‐up) is an 11‐item measure that includes the Satisfaction with Treatment Outcome and Treatment Experience domains. The BAS‐PP is a 2‐item measure for assessing the level of bother by appearance of vertical neck bands (Item 1) and jawline (Item 2), while the PGIS‐Jawline is a single‐item measure for assessing jawline definition. The ANLFQ: Impacts is a 7‐item measure for assessing psychosocial impacts.

The ANLFQ: Satisfaction (Baseline) was administered at baseline, the ANLFQ: Satisfaction (Follow‐up) was administered at follow‐up, and the other PROs were administered at all study visits. Additional details about each of the exploratory PRO scales and related endpoints are provided in Table 1 and reported previously [9].

### 2.4 Statistical Analysis

All analyses were performed using the modified intent‐to‐treat (mITT) population, defined as all randomized subjects who had ≥ 1 post‐baseline assessment of C‐APPS. PRO analyses using SAS version 9.3 (SAS, Cary, NC, USA) were performed for all visits based on the observed values without imputation for missing data (see Table 1 for a description of all measures). Descriptive statistics were used to summarize subject disposition, demographic characteristics, and PRO analyses. Responder rate analyses were performed for the ANLFQ: Satisfaction (Follow‐up), BAS‐PP, and PGIS‐Jawline. Two‐sided 95% confidence intervals (CIs) were calculated for both proportions of responders and difference in the proportion of responders based on the Wald method. *P‐*values were calculated from the Cochran–Mantel–Haenszel (CMH) tests for equality of responder proportions after adjusting for the stratification factor (baseline C‐APPS). The mean total scores were calculated for the ANLFQ: Satisfaction (Baseline) domains (Treatment Expectations and Condition‐related Satisfaction) and ANLFQ: Satisfaction (Follow‐up) domains (Treatment Outcome and Treatment Experience). The mean change from baseline was calculated for ANLFQ: Impacts. Two‐sided *t*‐tests were used to assess *P‐*values between groups, and 95% CIs were calculated. The PRO analyses were not included in the prespecified statistical hierarchy of the study hypotheses testing; therefore, unadjusted *P‐*values are presented for descriptive purposes to compare the effect of onabotA between each treatment group and placebo and are not intended for confirmatory inference. No adjustment for multiplicity was made; therefore, the results should not be interpreted as evidence of statistical significance.

**TABLE 1:** Patient‐Reported Outcomes Assessments and Endpoints.

PRO Scale	Description	Endpoints
ANLFQ: Satisfaction (Baseline)	7‐item measure to assess treatment expectations and satisfaction with the appearance of the neck and lower face at baseline	1. Mean total score for Treatment Expectations domain (range, 3–15). Lower scores indicate a higher level of expectation for improvement in the appearance of vertical neck bands, jawline, and skin firmness after treatment 2. Mean total score for Condition‐related Satisfaction domain (range, 4–20). Lower scores indicate greater satisfaction with the appearance of the neck and lower face
ANLFQ: Satisfaction (Follow‐up)	11‐item measure to assess satisfaction with treatment outcomes and experience with the treatment for the appearance of their neck and lower face	1. Mean total score for Satisfaction with Treatment Outcome domain (range, 6–30) 2. Mean total score for Satisfaction with Treatment Experience domain (range, 5–25). Lower scores indicate greater satisfaction with the appearance of the neck and lower face 3. Response rate (Item 5): proportion of subjects answering “*Satisfied*” or “*Very satisfied*” with effect of treatment
Bother Assessment Scale for Platysma Prominence (BAS‐PP)	2‐item measure to assess the level of bother by the appearance of vertical neck bands (Item 1) and jawline (Item 2) on a 5‐grade scale (1 = *Not at all bothered* to 5 = *Extremely bothered*)	1. Response rate (Item 1): proportion of subjects answering “*Not at all bothered*” or “*A little bothered*” 2. Response rate (Item 2): proportion of subjects answering “*Not at all bothered*” or “*A little bothered*”
Participant Global Impression of Severity (PGIS)‐Jawline	Single‐item measure to assess jawline definition before and after treatment on a 5‐grade scale (1 = *No sagging to* 5 = *Extreme sagging or drooping*)	1. Response rate: proportion of subjects answering “*No sagging or drooping*” or “*Mild sagging or drooping*” after treatment
Appearance of Neck and Lower Face Questionnaire (ANLFQ): Impacts	7‐item measure to assess the psychosocial impacts of neck and lower face appearance. The concepts assessed included appearance‐related impacts, emotional impacts, and social impacts of PP. Scores range from 7 (lowest impact) to 35 (highest impact)	1. Mean change from baseline in total score

## 3 Results

### 3.1 Patients

Of the 171 subjects who were randomized, 146 (85.4%) completed the study. The main cause for discontinuation was related to COVID‐19. Additional details of subject disposition are reported elsewhere [4]. The mITT population, defined as all randomized subjects who had ≥ 1 post‐baseline assessment of C‐APPS, was composed of 164 subjects with a mean age (range) of 50.0 (26–77) years (Table 2). The majority of subjects were female (95.1%) and White (93.9%); 10.4% of subjects were Latino or Hispanic. Demographic characteristics were comparable across the 3 treatment groups.

**TABLE 2:** Baseline Subject Characteristics.

	Placebo (*n* = 53)	OnabotA Dose 1^a^ (*n* = 58)	OnabotA Dose 2^b^ (*n* = 53)	Total (*N* = 164)
Age, mean (SD)	49.3 (9.6)	51.8 (9.2)	48.6 (10.2)	50.0 (9.7)
Female, *n* (%)	52 (98.1)	54 (93.1)	50 (94.3)	156 (95.1)
BMI, mean (SD), kg/m^2^	22.6 (2.6)	23.1 (3.0)	23.1 (2.7)	22.9 (2.8)
Race/Ethnicity, *n* (%)				
White	49 (92.5)	56 (96.6)	49 (92.5)	154 (93.9)
Hispanic or Latino	5 (9.4)	6 (10.3)	6 (11.3)	17 (10.4)
Black or African American	1 (1.9)	0 (0.0)	1 (1.9)	2 (1.2)
Asian	1 (1.9)	1 (1.7)	3 (5.7)	5 (3.0)
Native Hawaiian or Pacific Islander	2 (3.8)	1 (1.7)	0 (0.0)	3 (1.8)

*Note:* Table adapted with permission from Rohrich et al. [4].

Abbreviations: BMI, body mass index; onabotA, onabotulinumtoxinA; SD, standard deviation.

^a^ Dose 1 is either 26 U, 31 U, or 36 U onabotA.

^b^ Dose 2 is either 52 U, 62 U, or 72 U onabotA.

### 3.2 PRO Assessments

The following analyses were exploratory and were not included in the multiplicity‐controlled testing hierarchy. Thus, results, including unadjusted *P* values, should be interpreted descriptively.

#### 3.2.1 Satisfaction With Treatment

At baseline, the total scores on the ANLFQ: Satisfaction (Baseline) with Treatment Expectations domain (mean [SD], 8.2 [2.68] for placebo, 7.9 [2.66] for onabotA dose 1, and 7.5 [2.70] for onabotA dose 2) and Condition‐related Satisfaction domain (mean [SD], 16.2 [2.18] for placebo, 16.3 [2.37] for onabotA dose 1, and 17.0 [2.17] for onabotA dose 2) indicated that subjects had high levels of expectation that treatment would improve their PP and were less satisfied with the appearance of their neck and lower face at baseline, respectively. ANLFQ: Satisfaction (Baseline) scores were comparable across treatment groups.

**FIGURE 2:**
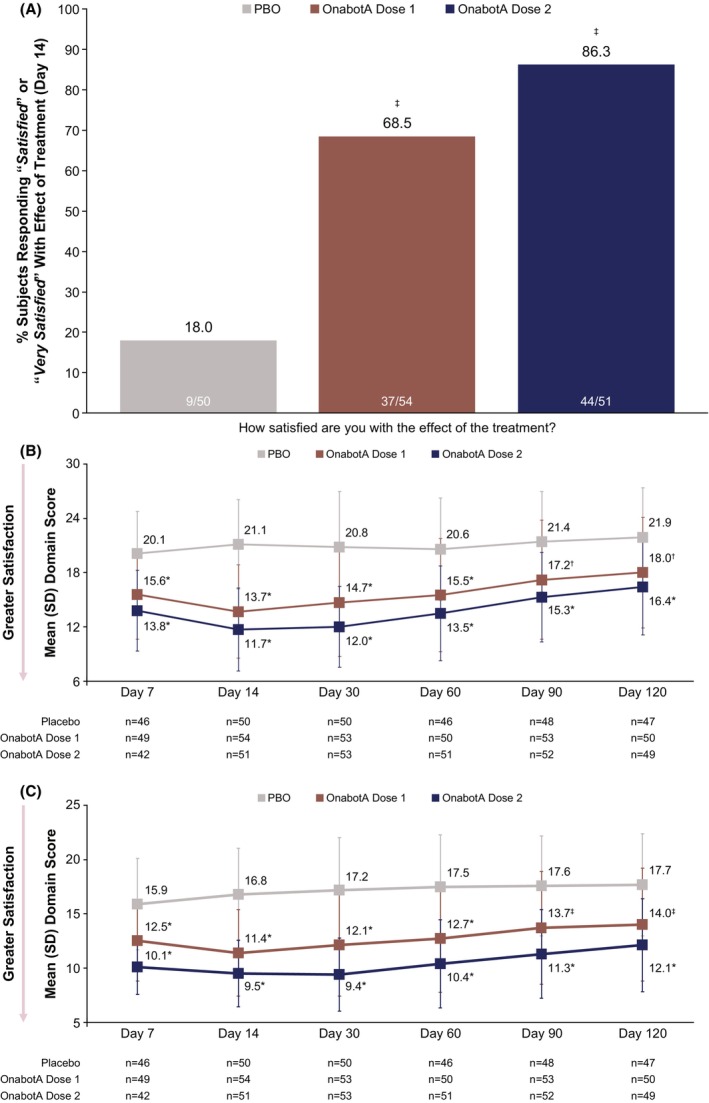
ANLFQ: Satisfaction (Follow‐up). (A) Responder rate for satisfaction with effect of treatment at day 14. Mean (SD) scores for (B) Satisfaction with Treatment Outcome domain and (C) Satisfaction with Treatment Experience domain. These assessments were evaluated in the mITT population. Dose 1 is either 26 U, 31 U, or 36 U onabotA, and dose 2 is either 52 U, 62 U, or 72 U onabotA. *P* values were not adjusted for multiplicity and were presented for descriptive purposes only. ANLFQ, Appearance of Neck and Lower Face Questionnaire; mITT, modified intent‐to‐treat; onabotA, onabotulinumtoxinA; PBO, placebo; SD, standard deviation. *p < 0.0001 vs. PBO. ^†^ *p <* 0.05 vs. PBO. ^‡^ *p* < 0.001 vs. PBO.

Posttreatment, a greater proportion of subjects reported being “*Satisfied*” or “*Very satisfied*” with the effect of treatment (Item 5) in each onabotA dose group compared with the placebo group at all timepoints. The response rate difference was ≥ 35.1 percentage points (95% CI: 17.9–52.3; unadjusted *p =* 0.0003) for onabotA dose 1 and ≥ 44.7 percentage points (95% CI: 27.9–68.1; unadjusted *p* = 0.0001) for onabotA dose 2 versus placebo at all timepoints. The greatest proportion of onabotA responders was observed at day 14 for both onabotA dose groups (Figure 2A).

The mean scores for the ANLFQ: Satisfaction (Follow‐up) with Treatment Outcome domain showed that subjects from both onabotA dose groups were more satisfied with their treatment outcome compared with subjects from the placebo group for all timepoints (unadjusted *p ≤* 0.0013), with the greatest effect at day 14 for each dose group (mean [SD] domain scores of 13.7 [5.1] in the dose 1 group and 11.7 [4.6] in the dose 2 group vs. 21.1 [5.0] for the placebo group; unadjusted *p* < 0.0001 for both groups; Figure 2B). Higher satisfaction with treatment outcomes in both onabotA dose groups versus placebo continued through day 120 (unadjusted *p* < 0.002 for both onabotA dose groups).

Mean scores for the ANLFQ: Satisfaction (Follow‐up) with Treatment Experience domain showed that subjects from each onabotA dose group were more satisfied with their treatment experience compared with subjects from the placebo group for all timepoints (unadjusted *p* ≤ 0.0005), with the greatest effect at day 14 for onabotA dose 1 (mean [SD] domain score of 11.4 [4.0]; unadjusted *p* < 0.0001) and at day 30 for onabotA dose 2 (mean [SD] domain score of 9.4 [3.4]; unadjusted *p* < 0.0001; Figure 2C). Higher satisfaction with treatment experience in both onabotA dose groups versus placebo continued through day 120 (unadjusted *p* ≤ 0.0005 for both onabotA dose groups).

#### 3.2.2 Bother With Vertical Neck Bands

**FIGURE 3:**
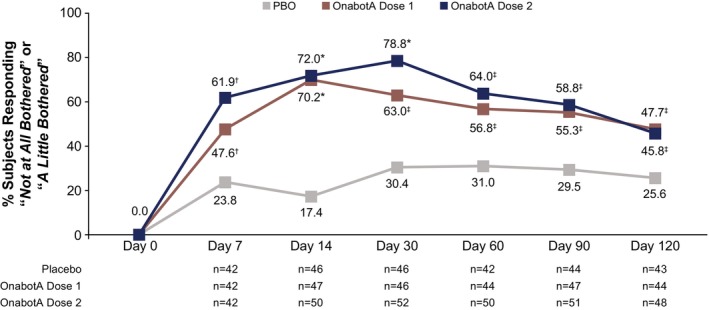
BAS‐PP Item 1 (Vertical Neck Bands) Responder Rates. These assessments were evaluated in the mITT population. Dose 1 is either 26 U, 31 U, or 36 U onabotA, and dose 2 is either 52 U, 62 U, or 72 U onabotA. *P* values were not adjusted for multiplicity and were presented for descriptive purposes only. BAS‐PP, Bother Assessment Scale for Platysma Prominence; mITT, modified intent‐to‐treat; onabotA, onabotulinumtoxinA; PBO, placebo. *p < 0.0001 vs. PBO. ^†^ *p* < 0.001 vs. PBO. ^‡^ *p <* 0.05 vs. PBO.

At baseline, most subjects (88.0% and 98.1% in the onabotA dose 1 and dose 2 groups, respectively, and 92.4% in the placebo group) were at least “*Somewhat bothered*” by the appearance of their vertical neck bands across all treatment groups. Among subjects who had reported they were at least “*Somewhat bothered*” by the appearance of their vertical neck bands at baseline, a greater proportion of subjects reported “*Not at all bothered*” or “*A little bothered*” following treatment in each onabotA dose group compared with the placebo group at all timepoints (Figure 3). The response rate difference was ≥ 22.1 percentage points (95% CI: 2.5–41.8; unadjusted *p* = 0.0332) for onabotA dose 1 and ≥ 20.3 percentage points (95% CI: 1.0–39.5; unadjusted *p* = 0.0460) for onabotA dose 2 versus placebo at all timepoints.

#### 3.2.3 Bother With Jawline

**FIGURE 4:**
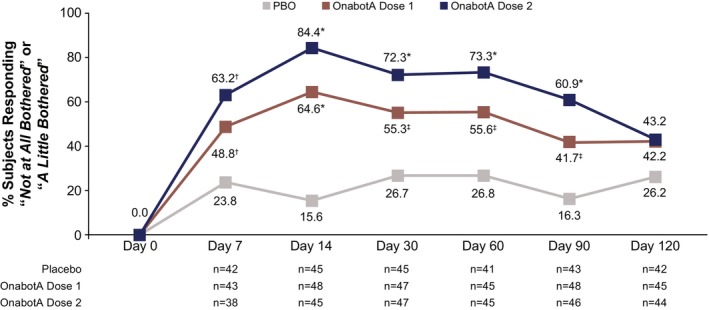
BAS‐PP Item 2 (Jawline) Responder Rates. These assessments were evaluated in the mITT population. Dose 1 is either 26 U, 31 U, or 36 U onabotA, and dose 2 is either 52 U, 62 U, or 72 U onabotA. *P* values were not adjusted for multiplicity and were presented for descriptive purposes only. BAS‐PP, Bother Assessment Scale for Platysma Prominence; mITT, modified intent‐to‐treat; onabotA, onabotulinumtoxinA; PBO, placebo. *p < 0.0001 vs. PBO. ^†^ *p* < 0.001 vs. PBO. ^‡^ *p <* 0.05 vs. PBO.

At baseline, most subjects (89.6% and 88.7% in the onabotA dose 1 and dose 2 groups, respectively, and 90.6% in the placebo group) were at least “*Somewhat bothered*” by the appearance of their jawline. Among subjects who had reported they were at least “*Somewhat bothered*” by the appearance of their jawline at baseline, a greater proportion of subjects reported “*Not at all bothered*” or “*A little bothered*” following treatment in each onabotA dose group compared with placebo at all timepoints (Figure 4). The response rate difference was ≥ 25.0 percentage points (95% CI: 5.3–44.8; unadjusted *p =* 0.0172) for onabotA dose 1 and ≥ 39.3 percentage points (95% CI: 19.3–59.4; unadjusted *p* = 0.0004) for onabotA dose 2 versus placebo at all timepoints up to day 90.

#### 3.2.4 Patient Perception of Jawline Definition

**FIGURE 5:**
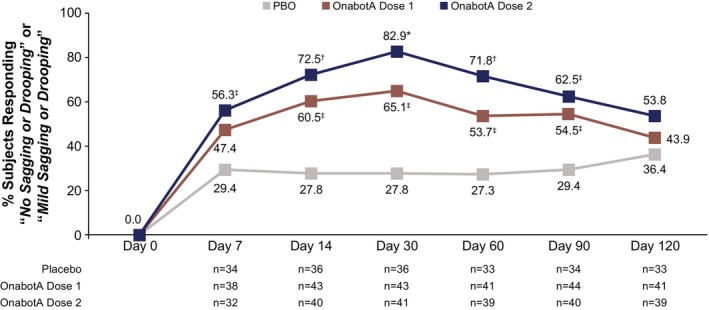
PGIS‐Jawline Response Rates. These assessments were evaluated in the mITT population. Dose 1 is either 26 U, 31 U, or 36 U onabotA, and dose 2 is either 52 U, 62 U, or 72 U onabotA. *P* values were not adjusted for multiplicity and were presented for descriptive purposes only. mITT, modified intent‐to‐treat; onabotA, onabotulinumtoxinA; PBO, placebo; PGIS, Participant Global Impression of Severity. *p < 0.0001 vs. PBO. ^†^ *p* < 0.001 vs. PBO. ^‡^ *p <* 0.05 vs. PBO.

The response rate difference was ≥ 25.1 percentage points (95% CI: 3.9–46.4; unadjusted *p =* 0.0274) for onabotA dose 1 from day 14 to day 90 and ≥ 26.8 percentage points (95% CI: 3.8–49.9; unadjusted *p* = 0.0287) for onabotA dose 2 from day 7 to day 90. For all other timepoints, the unadjusted *P* value was greater than 0.05 (Figure 5).

#### 3.2.5 Psychosocial Impacts Due to Platysma Prominence

At baseline, the mean ANLFQ: Impacts total scores were similar across the onabotA dose 1, onabotA dose 2, and placebo groups (22.4, 23.2, and 20.8, respectively). These scores indicated a higher psychosocial impact due to PP in all groups.

**FIGURE 6:**
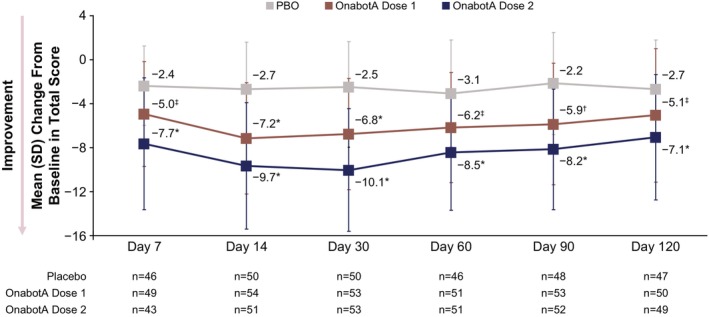
ANLFQ: Impacts. Mean (SD) change from baseline in ANLFQ total score. These assessments were evaluated in the mITT population. Dose 1 is either 26 U, 31 U, or 36 U onabotA, and dose 2 is either 52 U, 62 U, or 72 U onabotA. *P* values were not adjusted for multiplicity and were presented for descriptive purposes only. ANLFQ, Appearance of Neck and Lower Face Questionnaire; mITT, modified intent‐to‐treat; onabotA, onabotulinumtoxinA; PBO, placebo; SD, standard deviation. *p < 0.0001 vs. PBO. ^†^ *p* < 0.001 vs. PBO. ^‡^ *p <* 0.05 vs. PBO.

**FIGURE 7:**
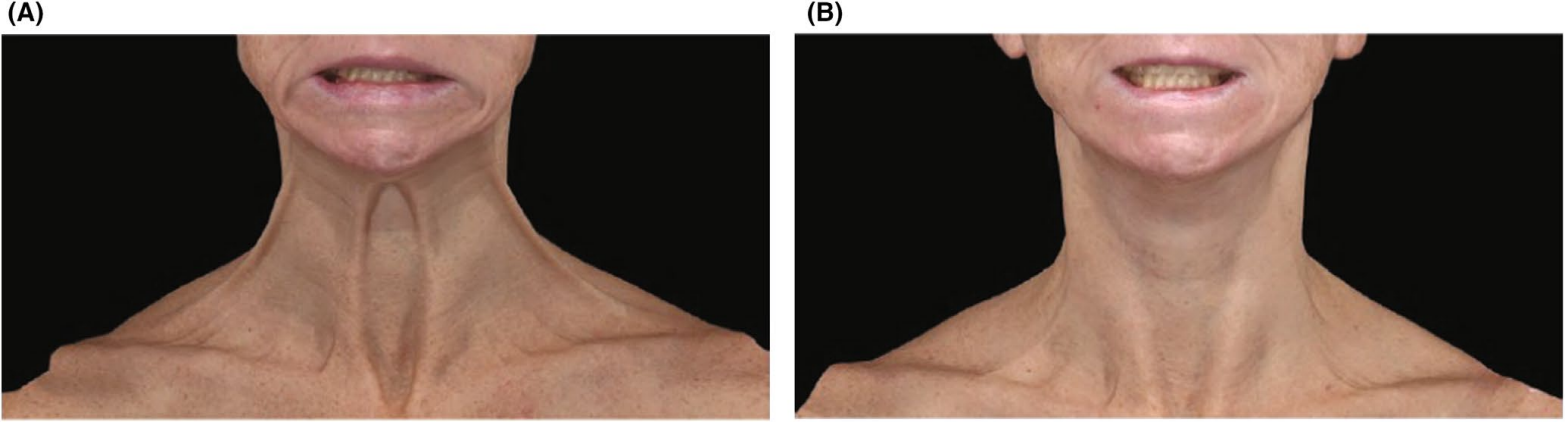
Representative images from a 42‐year‐old subject before and after treatment with onabotA. (A) Subject had grade 4 platysma prominence on both sides of the face at baseline based on the C‐APPS. (B) On day 14 after treatment with 36 U of onabotA, the subject achieved a 3‐grade improvement on the C‐APPS on both sides of the face. Reprinted with permission from Rohrich et al. [4]. C‐APPS, Clinician Allergan Platysma Prominence Scale; onabotA, onabotulinumtoxinA.

Posttreatment, the mean change from baseline in ANLFQ: Impacts total score was greater for both onabotA dose groups versus placebo across all timepoints (unadjusted *p ≤* 0.05; Figure 6), indicating improvement in the psychosocial impact of PP. The greatest change from baseline (mean [SD]) was observed at day 14 for onabotA dose 1 (−7.2 [5.09]) and at day 30 for onabotA dose 2 (−10.1 [5.6]). Representative images are shown in Figure 7.

### 3.3 Safety

The percentage of subjects reporting treatment‐emergent adverse events (TEAEs) was similar across the onabotA dose 1, onabotA dose 2, and placebo groups (23.7%, 25.9%, and 23.2%, respectively), with the most common TEAE being injection site bruising (6.8%, 5.6%, and 7.1%, respectively). Study drug–related TEAEs occurred in 0%, 1.7%, and 16.7% of subjects in the placebo, onabotA dose 1, and onabotA dose 2 groups, respectively; however, none resulted in study discontinuation. The onabotA dose 2 group included the most frequently reported study drug–related TEAEs of neck muscle weakness (9.3% [5/54]) and dysphagia (3.7% [2/54]). All neck muscle weakness events occurred in onabotA dose 2 subjects treated with 72 U (20%) and were mostly mild or moderate. However, 1 event was severe initially but improved over time. Dysphagia occurred in 1 subject each who were treated with 52 U (7.1%) and 62 U (6.7%); both events were mild and neither impaired functional swallowing.

Facial paresis (or mild depressor labii inferioris weakness) occurred in 1 subject each in the onabotA dose 1 group (26 U; 6.3%) and onabotA dose 2 group (52 U; 7.1%); both events were mild and resolved. Two unrelated serious adverse events (thrombocythemia and appendicitis) occurred in the onabotA dose 1 group. More detailed safety results are reported elsewhere [4].

## 4 Discussion

The prominence of the platysma muscle is a characteristic feature of the aging neck and is often regarded as aesthetically undesirable by affected individuals, impacting their social and emotional well‐being. The results of subject experiences from this study demonstrated that subjects in both onabotA dose groups reported higher satisfaction with treatment outcomes and treatment experience compared with subjects who received placebo. Subjects in both onabotA dose groups reported less bother from the appearance of their vertical neck bands and their jawline, less sagging or drooping of their jawline, as well as improved psychosocial impact related to their PP, compared with subjects who received placebo. The peak response rate across all measures was either on day 14 or day 30 for both onabotA dose groups, with a trend for improvement through day 120 versus placebo.

Because of the negative impact of PP on patients' quality of life, there is a need to assess experiences from the patient perspective to better understand the symptoms, impacts, and clinical burden of the condition, as well as treatment burden and patient preferences [10]. Regulatory agencies, such as the US Food and Drug Administration (FDA) and European Medicines Agency (EMA), have emphasized the importance of assessing patient experience in drug development research via detailed guidance for the industry on the development and use of PROs [8, 11, 12]. PROs are powerful tools that can better inform clinicians about the patient experience, including impact of their condition at baseline and how patients feel following treatment. Past clinical studies assessing PROs following treatment with botulinum toxin A for PP have reported improved appearance and quality of life, as well as a high satisfaction rate [13, 14, 15]. However, the PROs utilized were not specific to the assessment of PP, and included general aesthetic and quality of life assessment scales (e.g., the Investigators and Subjects Global Aesthetic Improvement Scale, the World Health Organization Quality of Life: Brief Version, and the Satisfaction and Self‐assessment Questionnaire) [13, 14, 15]. The current study is the first one to incorporate validated, fit‐for‐purpose PRO measures that were specifically developed to assess subjects' perceptions of the appearance of the neck and jawline and the impact of treatment on PP.

### 4.1 Study Strengths

The results of the study extend the observations of past studies describing the patient perspectives following treatment of PP with onabotA and have several notable strengths. For one, the current study uses validated PRO measures developed for the specific population of interest to evaluate patient experiences in a holistic manner, including their satisfaction with the effect of treatment, bother with vertical neck bands and jawline appearance, and improvements in jawline definition, as well as psychosocial impact. These PROs assess concepts that are important and relevant to those with PP because they were developed with direct input from individuals with PP. Furthermore, the PROs were validated rigorously per global regulatory and industry standards. In addition, the study findings reinforce the significant improvements in PP severity observed clinically after treatment in both onabotA dose groups [4] and suggest that these benefits further extend to improvements in subjects' overall perceptions of neck and jawline appearance as well as improvements in psychosocial impact, while maintaining high satisfaction with the effect of treatment.

### 4.2 Study Limitations

Similar to previously published results reported for the C‐APPS and P‐APPS [4], statistical comparisons between the onabotA dose groups were not evaluated, although there were dose‐dependent trends favoring the higher dose group (onabotA dose 2) over the lower dose group (onabotA dose 1). A study limitation is that the sample size may not be powered to detect differences between the dose groups. Additionally, analyses for the PROs were not prespecified in the testing hierarchy for hypothesis testing for this phase 2 study; therefore, all the results reported are based on unadjusted *P* values. Further confirmatory studies specifically designed and powered to evaluate these PRO assessments are warranted.

Another study limitation is that the majority of the subjects were White and female, thus limiting the generalizability and external validity of the findings to other racial/ethnic groups and genders. Differences in biological, cultural, psychosocial, or socioeconomic factors across groups may influence observed outcomes [16]. Therefore, our results may not reflect the experiences or preferences of underrepresented populations, and inadvertently emphasize aesthetic norms associated with the predominant demographic groups [16, 17]. Future studies with subjects from diverse racial, ethnic, and gender backgrounds are warranted to better understand how these factors may influence outcomes and to enhance the external validity of findings. Despite these limitations, our patient population is consistent with aesthetic toxin treatment seekers in real‐world settings [18, 19].

## 5 Conclusion

The results from the patient experience data to support the efficacy of treating moderate to severe PP with onabotA demonstrated high satisfaction with the effect of treatment, improved jawline definition and psychosocial impact, as well as reduced bother from the appearance of vertical neck bands and jawline.

## Author Contributions

Study investigator: David Bank, Brian Biesman, Shannon Humphrey, Joely Kaufman. Enrolled patients: David Bank, Brian Biesman, Shannon Humphrey, Joely Kaufman. Collection and assembly of data: Christy Harutunian, Warren Tong, Grace West, Vaishali Patel. Data analysis: Christy Harutunian, Warren Tong, Grace West, Vaishali Patel, Sandhya Shimoga. Data interpretation: All authors Manuscript review and revisions: All authors. Final approval of manuscript: All authors. All authors made substantial contributions to conception and design, acquisition of data, or analysis and interpretation of data; were involved in drafting the article or revising it critically for important intellectual content; provided final approval of the version to be published; and agreed to be accountable for all aspects of the work and ensuring that questions related to the accuracy or integrity of any part of the work are appropriately investigated and resolved.

## Funding

Allergan Aesthetics, an AbbVie company, funded this study and participated in the study design, research, analysis, data collection, interpretation of data, reviewing, and approval of the publication. All authors had access to relevant data and participated in the drafting, review, and approval of this publication. No honoraria or payments were made for authorship. Medical writing support was provided by Maria Lim, PhD and Jenna Bassett, PhD of Peloton Advantage LLC (an OPEN Health company) and funded by Allergan Aesthetics, an AbbVie company.

## Ethics Statement

The study was approved by an institutional review board (WIRB Copernicus Group [CGIRG], Cary, NC, USA) and was conducted in accordance with the International Council on Harmonization guidelines for Good Clinical Practice and the Council for International Organizations of Medical Sciences (CIOMS) Ethical Guidelines. All participants or their legally authorized representatives provided written informed consent.

## Conflicts of Interest

Shannon Humphrey is an investigator for Allergan Aesthetics (an AbbVie company). Warren Tong is a full‐time employee of Allergan Aesthetics (an AbbVie company) and current stockholder of AbbVie. Sandhya Shimoga, Grace S. West, and Vaishali Patel are full‐time employees of AbbVie and may own AbbVie stock. Joely Kaufman is an advisory board member for and receives research funding from AbbVie. David Bank has received funding as an investigator, consultant, and trainer for Allergan (an AbbVie company), Croma, Endo, Evolus, Galderma, and Revance. Brian Biesman receives research support from Allergan (an AbbVie company), Galderma, and Merz and is a consultant for Allergan (an AbbVie company), Galderma, Merz, and Revance. Christy Harutunian is a former employee and current stockholder of AbbVie as well as an employee of ASLAN Pharmaceuticals.

## Data Sharing

AbbVie is committed to responsible data sharing regarding the clinical trials we sponsor. This includes access to anonymized, individual and trial‐level data (analysis data sets), as well as other information (eg, protocols clinical study reports, synopses, or analysis plans), as long as the trials are not part of an ongoing or planned regulatory submission. These clinical trial data can be requested by any qualified researchers who engage in rigorous, independent scientific research, and will be provided following review and approval of a research proposal and Statistical Analysis Plan (SAP) and execution of a Data Use Agreement (DUA). Data requests can be submitted at any time after approval in the US and Europe and after acceptance of this manuscript for publication. The data will be accessible for 12 months, with possible extensions considered. For more information on the process, or to submit a request, visit the following link: https://vivli.org/ourmember/abbvie/ then select “Home.”

## Data Availability

Raw sequencing data and metadata files are available on figshare https://figshare.com/projects/MarsdenObjective1EthanolComparison/184573. All code with exact parameters and intermediary outputs used during bioinformatic processing, data curation, and statistical analysis are available on GitHub https://github.com/gjeunen/marsden_obj1_non‐destructive_heDNA. The GitHub repository will be provided with a Zenodo DOI upon acceptance of this manuscript.
